# The competency of nurses in basic life support at district hospitals in Cape Town, South Africa

**DOI:** 10.4102/safp.v67i1.6056

**Published:** 2025-05-29

**Authors:** George R. Mahembe, Robert Mash

**Affiliations:** 1 Division of Family Medicine and Primary Care, Faculty of Medicine and Health Sciences, Stellenbosch University, Cape Town, South Africa

**Keywords:** BLS, nursing, district hospital, knowledge, attitude, skill

## Abstract

**Background:**

Cardiac arrest (CA) is a leading cause of mortality, and survival rates are low; however, basic life support (BLS) can improve these rates. Nurses play a crucial role in the prevention, recognition, and response to CA, making BLS competency essential. Little is known about BLS competency among nurses in district hospitals. The study aimed to evaluate the knowledge, skills and attitudes of nurses regarding BLS in district hospitals in Cape Town, South Africa.

**Methods:**

A multi-centre, observational cross-sectional study was conducted. An existing self-administered questionnaire was adapted and validated. Stratified quota sampling selected 243 nurses. Data were analysed using the Statistical Package for the Social Sciences.

**Results:**

Only 3.0% of nurses scored above 80% on the knowledge test, while 15.9% scored that high on the skills test. Professional nurses, those with a bachelor’s degree, and nurses with any form of BLS training had significantly higher competency scores (*p* < 0.001). Basic life support certification rates were low at 50.8%, and certificates had expired in 52.5% of nurses. Furthermore, neither greater work experience (*r* = 0.025, *p* = 0.702) nor more frequent BLS performance (*p* = 0.083) was associated with higher competency scores.

**Conclusion:**

Nurses working in district hospitals demonstrated positive attitudes, but they had insufficient knowledge and poor BLS skills.

**Contribution:**

Nurses with higher-level qualifications and prior training in BLS achieved better competency scores. Attention should be directed towards CA registries, quality improvement systems, decentralised training at scale, performance management systems and an improved nurse staffing mix.

## Introduction

Cardiac arrest (CA) is one of the leading causes of mortality and remains a matter of global concern.^1^ It is a time-sensitive emergency with fatal sequelae; therefore, life-saving measures, such as basic life support (BLS), are crucial. Basic life support entails early recognition of CA, activation of emergency response, high-quality cardiopulmonary resuscitation (CPR) and early defibrillation.^2^ Positive outcomes for CA are dependent on organisational structures that support BLS as well as an appropriate intervention. Therefore, it is necessary that first responders and health systems are equipped to provide BLS without delay.

When CA occurs within the hospital, it is termed in-hospital cardiac arrest (IHCA), which has a global incidence of 0.5–10 cases per 1000 admissions.^3,4,5,6^ Generally, survival rates post-CA are low; however, research has shown the beneficial impact of BLS on survival.^7^ There are higher in-hospital survival rates of 46% reported in the paediatric population when compared to rates of approximately 15% in adults.^8,9^ One-year survival following IHCA is reported to be around 13%.^10^ Although these are all important factors, the evidence is largely from high-income countries and these pooled survival rates do not show variations between regions, hospitals, income levels and competency of health care providers (HCPs).^6,9,11^ Awareness of BLS may be particularly deficient in sub-Saharan Africa, with reported CPR attempt rates as low as 50% among first responders.^4^

In contrast to high-income nations such as the United States, Singapore, and Australia, where physician-centred health care systems are supported by advanced technology, low- and middle-income countries, such as South Africa, have far fewer human resources for health and rely more on nurses.^12^ This reliance, alongside a high disease burden, limited training opportunities and unequal distribution of health professionals between private and public sectors, significantly affects BLS.^12^ The country also contends with a persistent brain drain, as health care professionals, including nurses, migrate to higher-income countries, leading to a cascading impact that adversely affects patient care.^13^ In lower-income countries within the region, the challenges are similar but more severe because of poorer politico-economic conditions. These issues lead to inconsistencies in the quality of patient care, specifically BLS training and competency.

Nurses typically play crucial roles in the chain of survival for IHCA by recognising and responding to CA.^2^ Nurses may be first responders, resuscitation team members and clinical or administrative leaders.^14^ Typically, nurses are the first responders for IHCA through clinical nursing surveillance (CNS),^14,15^ and early detection is associated with improved outcomes.^6^ To fulfil this role, it is imperative that nurses have the awareness and capacity to detect and respond competently to CA.

Globally, there is a lack of CPR knowledge and skills among HCPs, particularly nurses.^16,17,18^ Findings in Africa align with this trend,^19,20,21^ with notably low BLS awareness, confidence and CPR attempt rates among HCPs.^4,22,23,24,25,26^ These studies do not provide any evidence from district health services in South Africa. The aim of this study was to evaluate the capability of nurses regarding BLS at district hospitals within the Khayelitsha-Eastern Sub-structure (KESS) of the Cape Town Metro Health Services.

## Research methods and design

A multi-centre observational descriptive cross-sectional study was implemented.

### Setting

The study was conducted in the Western Cape province of South Africa within the KESS. The KESS is one of four substructures in the metropolitan district of Cape Town, providing support services to clinics and hospitals in the Eastern and Khayelitsha subdistricts. It serves an estimated population of 1 200 211 people, of which 77% are uninsured and dependent on the public sector for health care.^27^ The study itself was conducted at three district hospitals within KESS: Helderberg, Eerste River, and Khayelitsha. Each of these hospitals had medical, surgical, paediatric, theatre, outpatient and emergency service points. However, only Helderberg Hospital and Khayelitsha Hospitals have obstetrics service points. Additionally, Helderberg Hospital is the only one of the three with a high-care unit. The types of nurses available at these hospitals ranged from professional nurse specialists to professional nurses, midwives, enrolled nurses (EN), and Enrolled nursing auxiliaries. Those who had speciality training had training in either primary care, emergency medicine, mental health, critical care, or perioperative nursing.

These nursing qualifications encompass both pre-2013 and post-2013 South African qualification frameworks. Before 2013, the South African nursing qualification framework consisted of four categories: (1) Enrolled nursing auxiliaries (ENA) requiring one year of training; (2) EN requiring two years of training; (3) registered nurses (RN) and/or midwives (M) requiring four years of training; and (4) specialist registered nurses and/or midwives necessitating one or two years of post-RN and/or M training. Post-2013, South Africa introduced five nursing qualifications leading to professional registration: (1) ENA with a one year higher certificate; (2) registered general nurses (RGN) with a three year diploma; (3) midwives with a one year advanced diploma; (4) professional nurse (PN) and/or professional midwife (PM) professional midwife with a four year bachelor’s degree; and (5) professional nurse specialists (PNS) and/or midwife specialists (PMS) with an additional one year postgraduate diploma.^13^ Consequently, the nursing workforce at our study sites included nurses with both legacy and new qualifications, reflecting the evolution of the qualification frameworks.

The South African Nursing Council (SANC) 2023 statistics indicated that 269 284 nurses were registered, with 89% being female, underscoring the gendered nature of the profession.^28^ The nurse ratios were 1:1.6:0.9 for EN:PN: PNS.^28^ However, this data did not differentiate between the public and private sectors, nor did it account for the distinction between nurses on the register and those actively practising. Approximately 18% of registered nurses are not in active practice, and around 60% work in the public sector, resulting in a low nurse density of about 400–500 per 100 000 population.^12,13^ Overall, this density is significantly lower compared to peer countries with similar metrics of economics, wealth distribution and population size.^12,13^ Hospital nurse-to-patient ratios are reported to range from 1:10 to 1:44.^29^

### Study population

The study population was defined as qualified nurses who were working within the medical, surgical, paediatric, obstetrics, theatre, emergency and high-care service points of the three hospitals. Nurses in administrative or managerial roles and nursing students were excluded.

### Sample size and sampling

It was assumed that 50% of participants would score at least 50% on a BLS knowledge test as this gave the largest sample size of at least 218 with a precision of 5%, 95% confidence intervals and a population size of 500.^21^ This sample size was inflated by 10% to 240, to account for missing data.

The researchers divided the sample size between Khayelitsha (51%), Helderberg (27%) and Eerste River (22%) hospitals in proportion to the number of beds as a proxy for the size of each hospital. The hospital’s sample was then evenly divided between available service points: (1) medical; (2) surgical; (3) emergency and high-care unit; (4) paediatric and neonatal; (5) obstetrics and gynaecology; (6) psychiatric; (7) operating theatre; and (8) outpatients and/or other.

### Data collection

Basic life support referred to both CPR and the use of an automated external defibrillator (AED), whereas CPR referred to external chest compressions and ventilation. A self-administered questionnaire was adapted from one previously used in the Philippine General Hospital to assess BLS in medical interns.^30^ The questionnaire was modified to align with the 2020 American Heart Association (AHA) BLS manual.^31^ Subsequently the tool was validated for this study with the help of an expert panel, comprised of two experts in BLS, and four on the local context and questionnaire design. The adapted tool was piloted with four nurses at Gustrouw Community Day Centre in KESS, with no further modifications required.

The questionnaire was divided into four sections: participant demographics; knowledge of BLS; BLS skills; and attitudes towards BLS. These sections contained questions on ventilation, sequence and delivery of CPR, depth, and rate of chest compressions as well as questions determining the age, sex, educational level, qualification, BLS training history and previous exposure to IHCA of participants. Questions on knowledge were presented as single-answer multiple-choice questions. Questions on skill were self-reported as ordinal categories (very important, important, and not important). Questions on attitude were answered on a four-point Likert scale from strongly agree (4) to strongly disagree (1).

On the day of data collection, the researchers visited each service point and coordinated with the nursing managers to establish the most suitable time and manner for engaging with the nurses on duty. The researchers then convened with the nurses, explained the objectives and aims of the research, and outlined the potential role of the nurses in the study. All nurses on duty that day were invited to participate. Interested nurses were provided with either an electronic or paper-based questionnaire, based on their preference. The questionnaires were completed under the supervision of the researchers to ensure quality control, with no time limit imposed for completion. The electronic questionnaire was designed using REDCap, with data automatically captured, while responses from the paper-based questionnaires were manually entered into REDCap by the principal investigator. Data collection occurred during June and July of 2023 and was repeated at each hospital until the required sample sizes were achieved.

### Data analysis

Questions on knowledge of BLS were given equal marks and scores out of 10 were converted to a percentage of correct answers. In line with the pass mark for acceptable knowledge of 84% in the American Heart Association (AHA) and/or Resuscitation Council of Southern Africa (RCSA) test, we report on those who achieved a score of 80% or more. Responses to questions on skill were scored out of 10 according to a selection of the most correct answers. Questions on attitudes were initially treated as ordinal variables without a numerical value, but for the purposes of measuring associations, the four-point Likert scale was treated as a numerical variable. Age, sex, educational level, qualification, years in practice, service point, BLS training history and previous exposure to IHCA were regarded as independent variables.

The Statistical Package for the Social Sciences (SPSS) was used for data analysis. We summarised categorical variables using frequencies and percentages, while numerical variables were summarised using means (standard deviations [s.d.]) or medians (interquartile ranges [IQR]) depending on the distribution. A one-way analysis of variance (ANOVA) was performed to analyse any association between education, training and professional rank on BLS competency. A composite score for BLS competency was created for inferential analysis by combining the scores for knowledge and skills to give a score out of 20. Spearman’s rank coefficient tested the association of years in practice and attitudes with BLS competency. A paired samples *t*-test was used to analyse the association of BLS certification and frequency of CPR on BLS competency.

### Ethical considerations

The study was approved by the Health Research Ethics Committee (HREC) and the Department of Health and Wellness of Stellenbosch University (S22/07/131) as well as from the Western Cape Government Health Strategy and Health Support on 08 March 2023 (WC_202212_023).

## Results

Table 1 summarises the characteristics of the 243 study participants. Participants were distributed between Eerste River (22.0%), Helderberg (30.3%), and Khayelitsha (47.7%) district hospitals. Their mean age was 42.6 (s.d. 10.0) years. In addition, most of these nurses were female (93.4%). Service points included the following wards: medical (13.3%); surgical (16.7%); emergency and high-care (13.4%); paediatric and neonatal (10.9%); obstetrics and gynaecology (12.5%); psychiatric (10.4%); operating theatre (12.5%); and outpatients and/or other (10.5%). The largest proportion of participants were ENA (37.9%). In contrast, the proportion of PNS accounted for only 9.6%. Nurses in the PNS group are specialised in critical care (5.0%), emergency care (10.0%), mental health (15%), midwifery (30.0%), perioperative care (25.0%), or primary care (15.0%). The median number of practice years was 10 (IQR 6–16) years. While 50.8% of participants reported having BLS certification, fewer than half (47.5%) possessed a valid certificate (obtained in the last two years).

**TABLE 1:** Characteristics of participants.

Characteristic	*n*	%
**Age group (years) (*N* = 238)**		
20–29	20	8.4
30–39	72	30.3
40–49	85	35.7
50–59	47	19.7
60–69	14	5.9
**Gender (*N* = 243)**		
Male	16	6.6
Female	227	93.4
**Level of education (*N* = 242)**		
Postgraduate Diploma in Nursing	20	8.3
Bachelor’s Degree in Nursing	29	12.0
Diploma in Nursing	78	32.2
Higher Certificate in Nursing	115	47.5
**Professional rank (*N* = 240)**		
Professional nurse with speciality	23	9.6
Professional nurse	59	24.6
Enrolled nurse	67	27.9
Enrolled nursing assistant	91	37.9
**BLS certification (*N* = 240)**	122	50.8
**BLS certification obtained (*N* = 122)**		
In the last six months	14	11.5
Between six months and one year ago	21	17.2
Between one year and two years ago	23	18.9
More than two years ago	64	52.5
**Any form of BLS training (*N* = 240)**		
Never	64	26.7
In the last six months	26	10.8
Six months to one year	21	8.8
Between one and two years	31	12.9
More than two years ago	98	40.8
**Frequency of CPR performance (*N* = 240)**		
Once a week	28	11.7
Once a month	35	14.6
Once every three months	31	12.9
Once every six months	12	9.2
Once a year	30	12.5
Not in the last year	94	39.2

BLS, basic life support; CPR, cardiopulmonary resuscitation.

Table 2 summarises performance in the knowledge and skill sections. Overall, only 34.6% had a knowledge score of > 50%, while 75.5% achieved a skills score of > 50%. If 80% is taken as a cut-off for acceptable knowledge, then only 3.0% achieved this threshold.

**TABLE 2:** Knowledge and skill scores.

Score	Knowledge scores out of 10 (*N* = 237)		Skill scores out of 10 (*N* = 233)	
	*n*	%	*n*	%
< 50%	155	65.4	57	24.5
50% – 79%	75	31.6	139	59.6
≥ 80%	7	3.0	37	15.9

An analysis of the knowledge and skill questions is depicted in Table 3. The most poorly answered knowledge questions were on the initial steps of BLS, the use of defibrillators, management of ventricular fibrillation and the qualities of good CPR. Although nurses scored better on skill than knowledge, both performances were poor. Nurses performed strongest on the timing of shock delivery, actions before and after AED arrival, and the rate and technique of chest compressions.

**TABLE 3:** Participants’ knowledge and skills of basic life support.

Variable	Correct responses	
	*n*	%
**Knowledge questions**		
What is the ratio of chest compressions to ventilation in an adult? (*N* = 239)	150	62.8
A woman sustained a potential cervical spine injury after a road crash. As a volunteer on the scene of the crash, what manoeuvre should be done on our patient to open the airway? (*N* = 240)	141	58.8
An elderly man complains of sudden chest pain and collapses on the floor. What is the best treatment for cardiac arrest until the arrival of advanced life support care? (*N* = 239)	138	57.7
What is the most successful treatment for ventricular fibrillation? (*N* = 239)	109	45.6
An elderly man complains of severe epigastric pain and collapses on the floor. What is the most frequent initial rhythm in witnessed cardiac arrest? (*N* = 239)	93	38.9
Which one of the following is *true* regarding the use of defibrillators? (*N* = 239)	91	38.1
A patient arrests on the operating table. Cardiopulmonary resuscitation (CPR) is immediately initiated. After two cycles, a rhythm check is performed and ventricular fibrillation is seen. Shock is delivered. What is the next step? (*N* = 238)	87	36.6
A 25-year-old male sustained a gunshot wound to the chest after having an encounter with the police. He goes into cardiac arrest as a result of his injuries. Which of the following is *false* regarding the basic life support (BLS)? (*N* = 239)	66	27.6
The emergency response team responds to a cardiac arrest in the ward. They should observe all of the following qualities of good CPR *except*? (*N* = 237)	59	24.9
A female nurse sees an older person with diabetes collapse in the middle of the parking lot at the hospital. What should be her first step in providing basic life support? (*N* = 240)	30	12.5
**Skill statements**		
I deliver the shock to the patient while others are still performing chest compressions. (*N* = 233)	181	77.7
I maintain my arms at a 90-degree angle with respect to the patient’s chest when I give chest compressions. (*N* = 234)	178	76.1
When I do chest compressions, I focus on getting the appropriate rate. (*N* = 234)	160	68.4
When I’m tired, I continue doing chest compressions without asking someone else to take over. (*N* = 234)	157	67.1
I continue CPR until the automated external defibrillator (AED) is turned on and the pads attached. (*N* = 234)	156	66.7
I push hard ensuring chest wall depression during chest compressions. (*N* = 234)	127	54.3
I give the next chest compression before completing the chest recoil. (*N* = 234)	106	45.3
I instruct a rescuer to push hard and fast whenever I see them slowing down on chest compressions. (*N* = 234)	105	44.9
I push with my whole body during CPR. (*N* = 234)	81	34.6
I count in my head when I do chest compressions. (*N* = 234)	69	29.5

CPR, cardiopulmonary resuscitation.

Table 4 summarises the attitudes of the participants towards BLS and their corresponding composite scores. In general, participants had positive attitudes towards BLS but felt that they needed more training, particularly for BLS in infants and in the use of the AED.

**TABLE 4:** Participants’ attitudes towards basic life support.

Statements	Mean score
I do not need skills in basic life support as it is rarely successful. (*N* = 236)	1.8
I am confident in the use of an automated external defibrillator (AED). (*N* = 236)	2.3
I think nurses have adequate training in basic life support. (*N* = 235)	2.4
I am confident in my knowledge and skills to perform basic life support on an infant (less than one year old). (*N* = 236)	2.5
I am comfortable performing basic life support on a child one year to eight years old. (*N* = 236)	2.6
I think I would be able to perform basic life support outside the hospital setting. (*N* = 236)	2.8
If faced with a situation requiring it, I am willing to provide basic life support within the hospital setting. (*N* = 236)	3.2
I feel that I need more training in doing basic life support. (*N* = 236)	3.3
I think basic life support should be taught to non-clinical health care workers like general assistants, cleaners and receptionists. (*N* = 236)	3.3
I think all nurses should have a review course on basic life support regularly. (*N* = 236)	3.5

Table 5 depicts the associations between the overall competency score and the characteristics of the participants. There was a statistically significant association between the level of education and BLS competency (*p* < 0.001). A post hoc analysis found that nurses with a Bachelor’s Degree in Nursing (BSN) had significantly higher BLS scores than those with a Diploma in Nursing (DN) or Higher Certificate in Nursing (HCN). Moreover, the study also found a significant association between BLS competency scores and professional rank (*p* < 0.001). A post-hoc analysis revealed that professional nurses (PN) significantly outperformed ENs and ENAs. There was also a significant association between BLS training and BLS competency scores (*p* < 0.001). Post hoc analysis found that nurses who ‘never’ had any form of BLS training performed significantly worse. While having a BLS certification conferred a statistically significant better performance (*p* = 0.001), there was no significant relationship between performing CPR frequently (*p* = 0.083) or the number of years in practice (*r* = 0.025, *p* = 0.702).

**TABLE 5:** Associations between overall competency score and characteristics of respondents.

Variables	BLS competency		
	Mean score	95% CI	*P*
**Level of education**	-	-	< 0.001
Postgraduate Diploma in Nursing	10.2	8.9–11.5	-
Bachelor’s Degree in Nursing	11.3	10.3–12.3	-
Diploma in Nursing	9.5	8.9–10.0	-
Higher Certificate in Nursing	8.7	8.2–9.3	-
**Professional rank**	-	-	< 0.001
Professional nurse with speciality	9.9	8.7–11.1	-
Professional nurse	10.9	10.3–11.6	-
Enrolled nurse	9.0	8.5–9.6	-
Enrolled nursing assistant	8.5	7.9–9.1	-
**Any form of BLS training**	-	-	< 0.001
Never	7.9	7.3–8.5	-
In the last six months	10.5	9.3–11.6	-
Six months to one year	10.3	9.1–11.4	-
Between one and two years	9.8	8.7–10.8	-
More than two years	9.8	9.2–10.3	-
**Frequency of CPR performance**	-	-	0.083
Once a week	8.8	7.8–9.7	-
Once a month	9.2	8.3–10.1	-
Once every three months	9.1	8.2–10.1	-
Once every six months	11	9.7–12.3	-
Once a year	9.1	7.8–10.3	-
Not in the last year	9.5	8.9–10.0	-
**BLS certification**	-	-	0.001
Yes	10.0	9.5–10.5	-
No	8.8	8.3–9.3	-

BLS, basic life support; CPR, cardiopulmonary resuscitation.

## Discussion

### Key findings

Despite having positive attitudes towards BLS, nurses in these district hospitals lacked adequate knowledge and skills to perform BLS effectively, which resonates with findings from tertiary hospitals. Professional nurses, nurses with a Bachelor’s degree and those with some form of BLS training had significantly higher competency scores in BLS (*p* < 0.001). No significant differences were found between professional nurses and those with a speciality nor between nurses holding a Bachelor’s degree and those with a Postgraduate Diploma in Nursing. Basic life support certification rates were low (50.8%), and in over half (52.5%) of the cases, the certification had expired. Nurses with such low BLS completion and certification rates had significantly lower BLS competency scores. Years of experience and performing BLS more frequently were not associated with better BLS competency scores.

### Discussion of key findings

Our study’s participants did not exhibit good knowledge of BLS, which contrasts with findings from other studies that demonstrated a strong theoretical understanding and retention of CPR knowledge among nurses following training.^16,17^ However, the intensity and frequency of formal training are crucial factors.^18^ In our study, more recent BLS certification and formal nursing training were associated with better competency scores. This observation aligns with previous research indicating that more formal educational programmes are associated with better knowledge.^16,17^ Importantly, our findings suggest that more recent training may have a greater impact on these outcomes than the possession of a degree alone.

Low rates of competency and certification have been recorded in other African settings and internationally, and in different levels of care.^17,18,21,22,23,24^ While we observed a positive association between BLS competency scores and training, other studies have not.^22,24^ However, these other studies assessed both BLS and Advanced Life Support. Advanced life support is a higher level of care that requires knowledge of drug and fluid management, endotracheal intubation, and trauma patient management. In addition, fewer nurses had recent training in these other studies. Nevertheless, most international literature reports similar results.^18^

The ageing nurse workforce could be an important factor, as older nurses require more regular updating of their competencies and are more indifferent to service adaptations that include training.^32,33^ This may partially account for the low prevalence of current BLS certifications, as over 60% of the nurses in our study were aged 40 years or older. Although senior professionals often benefit from extensive clinical exposure and ongoing professional development when such opportunities are available, it is important to recognise that increased experience may also foster complacency. The latter appears more pronounced in our sample, likely because of a pervasive lack of enforced training policies. Inadequate clinical mentoring, insufficient time to attend training, disruption of training because of the coronavirus disease 2019 (COVID-19) and the potential cost of training programmes are additional factors.^22,24^ Internationally, the nursing workforce tends to be relatively young and amenable to service adaptations.

Evidence suggests that health facilities with a higher proportion of nurses with BSN degrees have lower mortality rates and better outcomes of resuscitation.^34,35^ They are more competent in BLS and are more inclined to identify and act on clinical deterioration or CA. Our study suggests that this may also be the case locally as nurses with BSN degrees had higher competency scores. The PNs also had significantly better scores, but nurses with these more advanced qualifications and ranks made up a small proportion of the workforce. Nurses at a ratio of EN:PN: PNS equal to 1:0.9:0.4, was far below the recommended staffing norm of 1:1:1.^36^ A better staffing ratio could improve BLS and reduce in-hospital mortality.

While some studies have demonstrated that more experience and practising BLS led to greater retention of skills,^22^ this was not found here. This may be attributable to the fact that over half (54%) of the participants had their most recent exposure to CPR more than 1 year prior. In addition, there was a pervasive lack of initial training and valid certification among nurses. There also appeared to be a subset of nurses who believed erroneously that they did not need training or that BLS was not a skill worth acquiring. Exposure to real-life resuscitation scenarios and simulation exercises can enhance staff confidence and foster more positive perceptions of the skills required for BLS.^7^ Despite the substantial lack of training and valid accreditation, most nurses had positive attitudes towards BLS, underscored by a strong belief that nurses should participate in refresher courses. However, our study also uncovered concerns over the necessity of performing CPR, echoing findings by others who observed that contextual and regulatory factors in different regions shape HCP attitudes.^4^ Moreover, logistical and resource limitations in our setting may have reduced the frequency of practical training sessions, thereby adversely affecting BLS skill proficiency.^8,23^ These factors appear to be less problematic in well-resourced settings where streamlined protocols and institutional backing are more robust.

### Strengths and limitations

This study had an adequate sample size across multiple district hospitals. However, it had some limitations, such as the absence of an objective evaluation of BLS skills, relying instead on self-report. Associations should be interpreted with caution, as causality cannot be established. Although the questionnaire was sourced from a different context, it was adapted and validated locally, and aligned with current guidelines to ensure it was a reliable and valid instrument. We calculated a competency score from the individual knowledge and skill scores to test for associations with underlying variables. This score enabled inferential analysis but a threshold for competency was not established. A score of 80% or more on the knowledge test was considered a reasonable threshold based on the AHA/RCSA tests.

### Recommendations

Audit and feedback on the quality of resuscitation in CA is a requisite for quality improvement and strategies such as a CA registry can improve BLS.^37^ Given the established benefits of CA registries, the growing body of literature recommends hospitals participate in national registries.^5^ Unfortunately, there are no active resuscitation-focussed CA registries in Africa.^38^ We recommend that regions across Africa implement such measures as part of quality assurance, for example in South Africa this could be part of the Ideal Hospital initiative.^39^

As countries strive towards universal health coverage, the deployment of family physicians in district health services and their responsibility for clinical governance could enable BLS training at scale. The current centralised approach to BLS training limits capacity, and more decentralised training could improve coverage. Knowledge and skills also deteriorate within a year after training, which makes periodic retraining necessary.^17^ Health facilities should document the BLS certification status of nurses, and this can also be included in performance management systems. This guarantees that BLS training and competency are optimised and maintained at a consistent level.

Attention should also be given to the workforce, particularly the ratio of more to less qualified nurses. More highly trained and qualified nurses can also provide clinical mentoring to more junior colleagues.^40^

To gain a deeper understanding of the determinants influencing the implementation of BLS training and practice, future research may need to focus on more qualitative studies that explore the thoughts, experiences and opinions of HCPs.

## Conclusion

Nurses working in district hospitals had positive attitudes, but insufficient BLS knowledge and skills. Professional nurses, nurses with a degree and those with prior BLS training had better competency scores. Only half of the nurses had certification in BLS and only half of them had a valid certification. Years of experience and more frequent resuscitation experiences were not related to improved competency scores. Health services should consider the use of CA registries, more disseminated training opportunities, auditing of staff certification and performance management systems. Health systems should consider an appropriate staffing mix within the workforce, with more senior nurses, to support more successful and competent BLS.
